# Targeted Pten deletion plus p53-R270H mutation in mouse mammary epithelium induces aggressive claudin-low and basal-like breast cancer

**DOI:** 10.1186/s13058-015-0668-y

**Published:** 2016-01-19

**Authors:** Sharon Wang, Jeff C. Liu, Danbi Kim, Alessandro Datti, Eldad Zacksenhaus

**Affiliations:** Division of Advanced Diagnostics, Toronto General Research Institute - University Health Network, 67 College Street, Toronto, ON M5G 2M1 Canada; Laboratory Medicine & Pathobiology, University of Toronto, 1 King’s College Circle, Toronto, ON M5S 1A8 Canada; SMART Laboratory for High-Throughput Screening Programs, Lunenfeld-Tanenbaum Research Institute at Mount Sinai Hospital, 600 University Avenue, Toronto, ON M5G 1X5 Canada; Department of Agricultural, Food and Environmental Sciences, University of Perugia, Borgo XX Giugno, 74, 06121 Perugia, Italy

**Keywords:** Basal-like breast cancer, Claudin-low, p53 mutation, Pten, Metastasis, FDA-approved drugs, Mouse models

## Abstract

**Background:**

Triple-negative breast cancer (TNBC), an aggressive disease comprising several subtypes including basal-like and claudin-low, involves frequent deletions or point mutations in TP53, as well as loss of PTEN. We previously showed that combined deletion of both tumor suppressors in the mouse mammary epithelium invariably induced claudin-low-like TNBC. The effect of p53 mutation plus Pten deletion on mammary tumorigenesis and whether this combination can induce basal-like TNBC in the mouse are unknown.

**Methods:**

WAP-Cre:Pten^f/f^:p53^lox.stop.lox_R270H^ composite mice were generated in which Pten is deleted and a p53-R270H mutation in the DNA-binding domain is induced upon expression of Cre-recombinase in pregnancy-identified alveolar progenitors. Tumors were characterized by histology, marker analysis, transcriptional profiling [GEO-GSE75989], bioinformatics, high-throughput (HTP) FDA drug screen as well as orthotopic injection to quantify tumor-initiating cells (TICs) and tail vein injection to identify lung metastasis.

**Results:**

Combined Pten deletion plus induction of p53-R270H mutation accelerated formation of four distinct mammary tumors including poorly differentiated adenocarcinoma (PDA) and spindle/mesenchymal-like lesions. Transplantation assays revealed highest frequency of TICs in PDA and spindle tumors compared with other subtypes. Hierarchical clustering demonstrated that the PDA and spindle tumors grouped closely with human as well as mouse models of basal and claudin-low subtypes, respectively. HTP screens of primary Pten^∆^:p53^∆^ vs. Pten^∆^:p53^R270H^ spindle tumor cells with 1120 FDA-approved drugs identified 8-azaguanine as most potent for both tumor types, but found no allele-specific inhibitor. A gene set enrichment analysis revealed increased expression of a metastasis pathway in Pten^∆^:p53^R270H^ vs. Pten^∆^:p53^∆^ spindle tumors. Accordingly, following tail vein injection, both Pten^∆^:p53^R270H^ spindle and PDA tumor cells induced lung metastases and morbidity significantly faster than Pten^∆^:p53^∆^ double-deletion cells, and this was associated with the ability of Pten^∆^:p53^R270H^ tumor cells to upregulate E-cadherin expression in lung metastases.

**Conclusions:**

Our results demonstrate that WAP-Cre:Pten^f/f^:p53^lox.stop.lox_R270H^ mice represent a tractable model to study basal-like breast cancer because unlike p53 deletion, p53^R270H^ mutation in the mouse does not skew tumors toward the claudin-low subtype. The WAP-Cre:Pten^f/f^:p53^lox.stop.lox_R270H^ mice develop basal-like breast cancer that is enriched in TICs, can readily form lung metastasis, and provides a preclinical model to study both basal-like and claudin-low TNBC in immune-competent mice.

**Electronic supplementary material:**

The online version of this article (doi:10.1186/s13058-015-0668-y) contains supplementary material, which is available to authorized users.

## Background

Breast cancer is a highly heterogeneous disease comprising estrogen receptor alpha (ERα)-positive and HER2/ERBB2/NEU-positive subtypes as well as triple-negative breast cancers (TNBCs) that do not express ERα, human epidermal growth factor 2 (HER2) or the progesterone receptor. TNBC can be further divided into several additional groups including basal-like and mesenchymal/claudin-low [1–5]. These tumors contain mutations or deletion in the tumor suppressor p53 in 60–80 % of cases [4]. In addition, 35 % of TNBC also show loss of expression of the tumor suppressor phosphatase and tensin homolog deleted in chromosome 10 (PTEN). The latter is a phosphatase that converts phosphatidylinositol (3, 4, 5)-trisphosphate (PIP3) into phosphatidylinositol (4, 5)-disphosphate (PIP2), thereby antagonizing phoshotidylinositol-3 kinase (PI3K) pathway activation [6–8]. On the basis of frequency in which each gene is disrupted alone, combined loss of p53 and Pten in TNBC is calculated at about 21–28 %. In addition, using Pten RNA expression and p53 pathway activity it was estimated that 24.4 % of TNBCs are Pten-low, 65.6 % are p53 activity-low, and 18.7 % are both Pten-low and p53 pathway activity-low [9].

The generation of mice with mutations that occur in breast cancer offers a window into the mechanisms of tumor initiation, progression and spread in immune-competent mice, and provide preclinical models to test potential new therapies. Deletion of p53 in the mouse induces diverse tumors as well as claudin-low-like TNBC [10]. We recently showed that combined deletion of p53 and Pten via mouse mammary tumor virus (MMTV)-Cre^NLS^ or whey acidic protein (WAP)-Cre drivers induces almost exclusively claudin-low TNBC-like tumors [9]. This suggests that p53 deletion in the mouse promotes mesenchymal-like cancer, which is further accelerated by disruption of Pten.

To generate a model for basal-like Pten/p53 mutant TNBC, we here tested the effect of deleting Pten and expressing p53^R270H^ mutation in the DNA-binding domain (DBD) [11]. This and similar mutations in p53 DBD were shown to act as dominant-negative or gain-of-function alleles that accelerate metastasis through multiple mechanisms [12]. Targeted expression of p53^R270H^ in the mammary epithelium was reported to induce divergent tumors [13]. Here we show that WAP-Cre:Pten^f/f^:p53^lox.stop.lox_R270H^ composite mice develop spindle-like and poorly differentiated adenocarcinoma (PDA) as well as other subtypes. Using microarray profiling, we found that the spindle and PDA tumors clustered with claudin-low and basal-like breast cancer (BLBC), respectively, and metastasized to the lung following tail vein injection significantly faster than tumors from WAP-Cre:Pten^f/f^:p53^f/f^ double-deletion mice. Thus, WAP-Cre:Pten^f/f^:p53^lox.stop.lox_R270H^ mice provide a preclinical model to study aggressive Pten/p53-mutant basal-like and claudin-low TNBC.

## Materials and methods

### Mice

Animal protocols were approved by the University Health Network in accordance with the guidelines of the Canadian Council of Animal Care. WAP-Cre mice [14] kindly received from Dr. Lothar Hennighausen, National Institutes of Health (NIH), were crossed with Pten^f/f^ mice [15] from Dr. Tak Mak, Princess Margaret Cancer Centre (PMCC), as described [9], and then further mated with p53^LSLR270H/+^ mice [11], which were generated by Dr. Tyler Jacks, Massachusetts Institute of Technology (MIT), and obtained through National Cancer Institute (NCI) Mouse Models of Human Cancers Consortium (MMHCC). Compound mutant mice used in this study were on a mixed genetic background.

### PCR genotyping and deletion analysis

Compound mutant mouse strains utilized in this study were genotyped as previously described [16, 17]. Deletion of the Pten^f/f^ allele was detected by polymerase chain reaction (PCR) using the following primers: Forward: 5′GTCACCAGGATGCTTCTGAC3′, Reverse: 5′ACTATTGAACAGAATCAACCC3′ where Pten^f/f^ results in a 335 bp product and Pten^Δf/f^ in an 849 bp product. For p53^R270H^ recombination detection, the following primers were used to yield a wild-type band of 290 bp and mutant allele of 330 bp: wt Forward: 5′-TTACACATCCAGCCTCTGTGG-3′; mutant Forward: 5′-AGCTAGCCACCAT GGCTTGAGTAAGTCTGCA-3′; Reverse: 3′-CTTGGAGACATAGCCACACTG-3′. To determine the presence of the LSL cassette in p53, the following primers were used to detect a wild-type band of 166 bp and mutant allele of 270 bp: Forward: 5′-AGCCTGCCTAGCTTCCTCAGG-3′; Reverse: 5′-CTTGGAGACATAGCCACACTG-3.

### Primary tumor cell isolation

Tumor tissue was minced with sterile razor blades and placed in 100 U/ml collagenase/hyaluronidase solution (StemCell Technology, Vancouver, BC, Canada; number 07912) overnight at 4 °C, and then for 30 min at 37 °C with occasional mixing. Digested tissues were rinsed with Hank’s balanced salt solution (Gibco, Carlsbad, CA, USA) containing 2 % fetal bovine serum (FBS) (HF) and centrifuged at 1500 rpm for 5 min. Tumor cells were passed through a 40 mm cell strainer.

### Tumorsphere culturing in vitro

Cell suspension of single cell mammary tumor epithelial cells were plated onto ultra-low attachment plates (Corning, Tewksbury, MA, USA; Costar number 3473) in Dulbecco’s modified Eagle’s medium (DMEM)/F-12 Ham medium (Sigma-Aldrich, St. Louis, MO, USA; number D8900) containing 20 ng/ml basic fibroblast growth factor (bFGF) (Sigma-Aldrich; number F0291), 20 ng/ml epidermal growth factor (EGF) (Sigma-Aldrich; number E9644), 4 μg/ml of heparin (Sigma-Aldrich; number H4784) and B-27 supplement (1:50 dilution, Gibco; number 17504-044), and cultured at 37 °C; 5 % CO_2_ as described [18]. Spheres were mechanically and enzymatically dissociated weekly in 0.25X (0.05 %) Trypsin-EDTA solution (Gibco; number 25300) for 3 min at 37 °C, followed by gentle trituration for 1 min. To create tumor-derived primary cell lines, tumorspheres were plated in tissue culture plates and expanded under adherent conditions.

### Orthotopic transplantation and tail vein injection

Cells were transplanted into the number 4 mammary glands of 3–5-week-old immune-deficient NOD/SCID female mice as described [19]. Indicated number of cells were resuspended in 10 μl of media and mixed at 1:1 ratio with 10 μl matrigel (BD Biosciences, Franklin Lakes, NJ, USA; number 356234) on ice. Samples (total 20 μl) were then injected into mice under isoflurane anesthesia. Mice were monitored for tumor formation for up to 6 months. For tail vein injections, tumor-derived cell lines were dissociated into single cells and 200 μl of 1 × 10^5^ cells/100 μl resuspended in DMEM media injected into the lateral tail vein.

### Histology and immunohistochemistry

Paraffin sections (5 μm thick) were immunostained using the following antibodies/dilution: p53 (1:200; FL-393; Santa Cruz Biotechnology Inc., Dallas, TX, USA; number sc-6243), Ki67 (1:150; Biocare Medical Inc., Concord, CA, USA; number CRM325, clone SP6), CK6 (1:200, Covance, Princeton, NJ, USA; number PRB-169B), CK5 (1:20; NeoMarkers, Fremont, CA, USA; number XM26), CK14 (1:200; Panomics, Fremont, CA, USA; number E2624), CK18 (1:200; Fitzgerald Industries International, Acton, MA, USA number RDI-PR061028), and vimentin (1:50; Santa Cruz Biotechnology Inc.; number SC32322) as described [17]. Secondary biotinylated anti-mouse or anti-rabbit antibodies (Vector Laboratories, Burlingame, CA, USA) were used at 1:200 dilution. Immunohistochemistry (IHC) was performed using VECTASTAIN ABC Systems (Vector Laboratories). Sections were counterstained with methylene green.

### RNA isolation, microarray and cluster analysis

Total RNA was extracted using TRIZOL reagent (Invitrogen, Burlington, ON, Canada). Microarray analysis was carried out using Affymetrix Mouse Gene 1.0 ST with 500 ng of total RNA (Centre for Applied Genomics, Hospital for Sick Children, Toronto, ON, Canada), and data deposited in the Gene Expression Omnibus (GEO) under the accession number GEO:GSE75989. Microarray data were normalized using the robust multiarray averaging (RMA) method via Partek and log2-transformed gene expression values were obtained as described [19]. To compare our mouse models with human breast cancer (BC) and other published models, median-centered values of our mouse microarray data were integrated with GSE18229 (human BC) and GSE3165 (mouse models) by “distance-weighted discrimination” (DWD). The subtypes of human BCs in GSE18229 were predetermined with PAM50 and the claudin-low signature [9, 20, 21]. The DWD-integrated samples were classified by unsupervised hierarchical clustering (complete linkage) using shared intrinsic genes, the basal, luminal, and mesenchymal gene sets [20], and the claudin-low signature [21]. Values of gene expression were log2-transformed, median-centered, and visualized as heat maps by Java TreeView.

### GSEA pathway analysis

Gene expression data were analyzed using the gene set enrichment analysis (GSEA) Preranked method from the Broad Institute (version 2.2.0, [22]) with parameters set to 1000 gene set permutations. Genes were ordered using the “limma” package in R to obtain *t* values corresponding to each pair-wise comparison with Benjamini and Hochberg's method to control the false discovery rate (FDR). The gene sets included in the GSEA analyses were obtained from MSigDB containing BioCarta, KEGG, Matrisome, Pathway Interaction Database, Reactome, SigmaAldrich, Signaling Gateway, Signal Transduction KE, SuperArray gene sets (c2, v5.0) and the Gene Ontology (GO) databases (c5, v5.0), updated on March 2015 (http://www.broadinstitute.org/gsea/downloads.jsp). An enrichment map (version 1.1 of Enrichment Map software [23], was generated by Cytoscape (version 2.8.3) for each comparison using enriched gene sets with a nominal *p* value < 0.005, FDR < 0.1, and the overlap coefficient set to 0.5.

### Prestwick (FDA-approved) drug screen

High-throughput (HTP) screens of 1120 Food and Drug Administration (FDA)-approved drugs using a robotic Biomek FX liquid handler equipped with a pintool for automated compound dispensing were performed at the S.M.A.R.T facility, Samuel Lunenfeld Research Institute as previously described [9]. Cells were seeded onto 384-well plates, and pinned with chemicals resuspended in DMSO to reach a final concentration of 1 μM (Prestwick library, Prestwick Chemical, San Diego, CA, USA). As a reference for 100 % activity, each plate included wells with cells treated with vehicle only, and background was measured with media in the absence of cells. Alamar blue (Invitrogen) was added 2 d post-treatment, and cell viability was read 5 h later. Screen data were normalized by Z score [24].

### MTT viability assay

For validation experiments, cells were seeded in 96-well plates and treated the following day. At 3 d post-treatment, 20 μL of 2 mg/mL MTT (3-[4,5-dimethylthiazol-2-yl]-2, 5-diphenyl tetrazolium bromide, Sigma-Aldrich) were added and incubated for 4 h. MTT/media solution was aspirated and replaced with DMSO and cell viability was read after 5 min incubation.

### Additional statistical analysis

Survival, drug response and IHC data were analyzed using Mantel-Cox test, non-linear regression and Student’s *t* test, respectively. Tumor-initiating cell (TIC) frequency was calculated using L-cal (www.stemcell.com).

## Results

### Targeted Pten deletion plus expression of a p53-R270H mutant in mammary epithelium accelerate mammary tumorigenesis

**Fig. 1:**
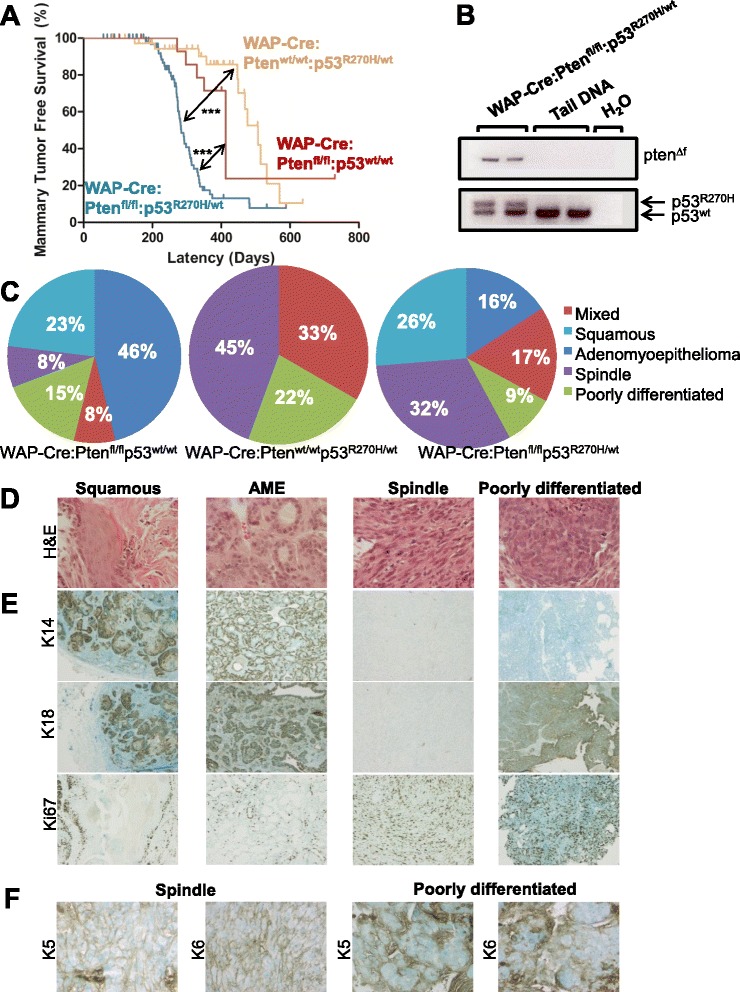
Pten deletion plus p53-R270H point mutation cooperate to accelerate mammary tumor formation. **a** Kaplan-Meier mammary tumor-free curves for WAP-Cre:Pten^fl/fl^:p53^wt/wt^ (n = 16; median latency - 413 days), WAP-Cre:Pten^wt/wt^:p53^R270H/wt^ (n = 39; median latency - 507 days), and WAP-Cre:Pten^fl/fl^:p53^R270H/wt^ (n = 75; median latency - 291 days) mice. Statistical significance by Wilcoxon method: Pten versus p53, *p* = 0.0118; Pten/p53 vs. Pten, *p* < 0.0001; Pten/p53 vs. p53, *p* < 0.0001. **b** Detection of conditional Pten deletion and p53 mutant allele by PCR. **c** Distribution of tumor types (%) in WAP-Cre:Pten^fl/fl^:p53^wt/wt^ (*left*; n = 13), WAP-Cre:Pten^wt/wt^:p53^R270H/wt^ (*middle*; n = 9), and WAP-Cre:Pten^fl/fl^:p53^R270H/wt^ (*right*; n = 76) mice. **d** Histology of the four major tumor types from WAP-Cre:Pten^fl/fl^:p53^R270H/wt^ mice. Original magnification 40×. Larger images are presented in Additional file 1. **e** Representative IHC analysis of basal differentiation marker (K14), luminal differentiation marker (K18) and proliferation marker ki67 in the major tumor types from WAP-Cre:Pten^fl/fl^:p53^R270H/wt^ mice. **f** Representative expression of basal differentiation marker (K5 and K6) in PDA (n = 7) and spindle (n = 3) tumors. *IHC* immmunohistochemistry, *PCR* polymerase chain reaction, *PDA* poorly differentiated adenocarcinoma

WAP-Cre:Pten^f/f^:p53^lox.stop.lox_R270H/+^ composite mice (WAP-Cre:Pten^f/f^:p53^lsl_R270H/+^ for short) developed mammary tumors with significantly shorter latency than parental WAP-Cre:Pten^f/f^ or WAP-Cre:p53^lsl_R270H/+^ females (Fig. 1a). Deletion of Pten and the loxP-stop-loxP cassette was confirmed by PCR (Fig. 1b). Histology analysis revealed that WAP-Cre:p53^lsl_R270H/+^ mice developed three major tumors types: spindle-like, PDA and mixed subtypes (Fig. 1c; Additional file 1). Tumor distribution in WAP-Cre:Pten^f/f^:p53^lsl_R270H/+^ mice was more similar to WAP-Cre:Pten^f/f^ mice including spindle-like, PDA, squamous carcinoma, adenomyoepitheliomas (AME) and mixed tumors. However, the ratio of these tumors varied (Fig. 1c). The spindle and PDA tumors were highly proliferative relative to other types as judged by Ki67 expression and expressed low levels of the myoepithelial cytokeratin marker, K14 (Fig. 1d). In contrast, the spindle tumors did not express the luminal marker, K18, whereas the PDAs showed low/variable expression pattern. We specifically stained the PDA and spindle-like tumors for the basal keratins K5 and K6, which are hallmarks of BLBC. We found that five of seven PDA were positive for K5, and four of seven were positive for K6. For the spindle tumors, three of three were positive for both K5 and K6 (Fig. 1e).

**Fig. 2:**
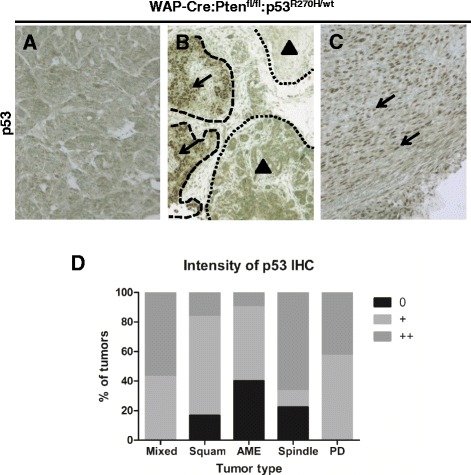
Analysis of mutant p53 accumulation in tumor cells. **a**-**c** p53 IHC of WAP-Cre:Pten^fl/fl^:p53^R270H/wt^ tumors showing lack of p53 accumulation, indicating the p53^R270H^ allele was not activated in (**a**). *Arrows* and *dashed outlines* indicate a region with strong p53 accumulation; *arrowheads* and *dotted outlines* to regions with weak or no p53 accumulation (**b**). Note strong nuclear accumulation of mutant p53 in tumor cells (**c**). **d** Summary of mutant p53 accumulation in the major tumor types in WAP-Cre:Pten^fl/fl^:p53^R270H/wt^ mice: mixed, n = 9; squamous, n = 4; AME, n = 9; spindle, n = 12; PDA, n = 6. *AME* adenomyoepithelioma, *IHC* immmunohistochemistry, *PDA* poorly differentiated adenocarcinoma

Prior to Cre-mediated recombination, the non-recombined p53^lsl_R270H^ gene acts as a null (−) allele [11], and the p53^lsl_R270H/wt^ mice are functionally p53^−/+^ in all tissues (including mammary epithelium). To determine if the p53^lsl_R270H^ allele was induced through deletion of the Lox-stop-lox cassette via WAP-Cre, we immunostained tumor sections for p53, which is stabilized by the p53^R270H^ mutation, leading to high expression. Some tumors were uniformly positive, indicative of Cre-recombination and p53^R270H^ expression; other tumors were negative or contained areas of p53-positive cells alongside p53-negative cells, likely reflecting clonal evolution (Fig. 2a-d). For all experiments (including IHC in Fig. 1) we only used and compared tumors with high p53 expression (i.e. expressing the p53^R270H^ allele) in large areas or throughout the tumor (e.g. Fig. 2c).

### Frequent tumor initiating cells (TICs) in poorly differentiated adenocarcinomas and spindle-like Pten^∆f^:p53^R270H^ tumors

**Fig. 3:**
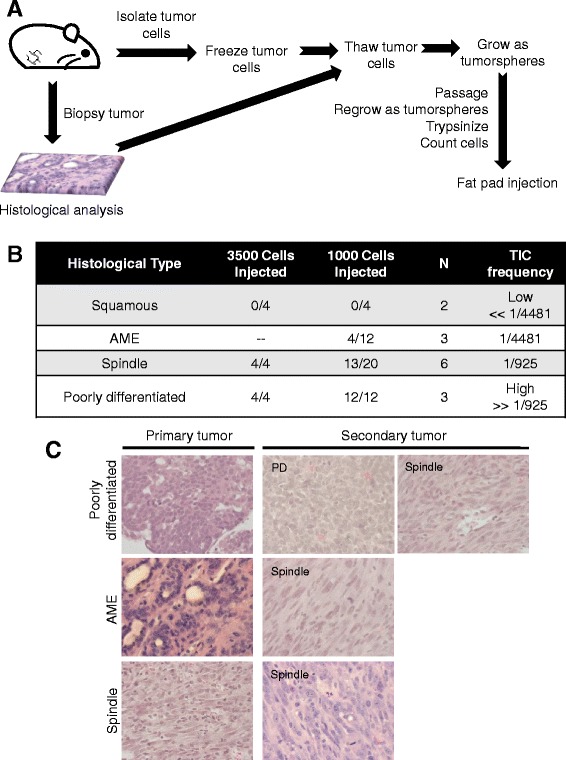
High frequency of TICs in PDA and spindle tumors. **a** Schematic illustration of the experimental design used to analyze TIC frequency in the four major tumor types. **b** Tumor-initiating cell (TIC) frequency in the major tumor subtypes in WAP-Cre:Pten^fl/fl^:p53^R270H/wt^ mice, showing high frequency in PDA and spindle tumors. **c** Histology of secondary tumors showing a selection for spindle-like tumors. For PDAs - 5/16 secondary tumors remained PDAs, and the rest (11/16) became spindle. For AME tumors, 4/4 secondary were all spindle-like. All primary spindle tumors gave spindle secondary tumors. *AME* adenomyoepithelioma, *PDA* poorly differentiated adenocarcinoma

One measure for tumor aggressiveness is the frequency of tumor-initiating cells (TICs), capable of inducing secondary tumors following transplantation into recipient mice [25, 26]. Pten^∆f^:p53^R270H^ tumors from each of the four different subtypes were dissociated to obtain single cells, cultured as tumorspheres in ultra-low attachment plates in serum-free media supplemented with EGF and FGF, passaged once, and then 1000 or 3500 cells were injected orthotopically into recipient female mice (Fig. 3a). While the squamous and AME gave undetectable or low TIC frequency of 1/4481, respectively, the spindle tumors showed TIC frequency of 1/925 and the PDA tumors an even higher frequency that was not calculatable because all recipient mice developed secondary lesions (Fig. 3b). Thus, Pten^∆f^:p53^R270H^ spindle and PDA tumors exhibit high TIC frequency, indicative of aggressive cancers. Interestingly, histology analysis of secondary tumors revealed dominant spindle histology even when primary lesions were from AME (4/4) or PDAs (11/16) (Fig. 3c), suggesting that they arose from rare mesenchymal/spindle subclones within these lesions.

### Poorly differentiated and spindle-like Pten^∆f^:p53^R270H^ tumors cluster with known mouse models of basal-like and claudin-low breast cancer

**Fig. 4:**
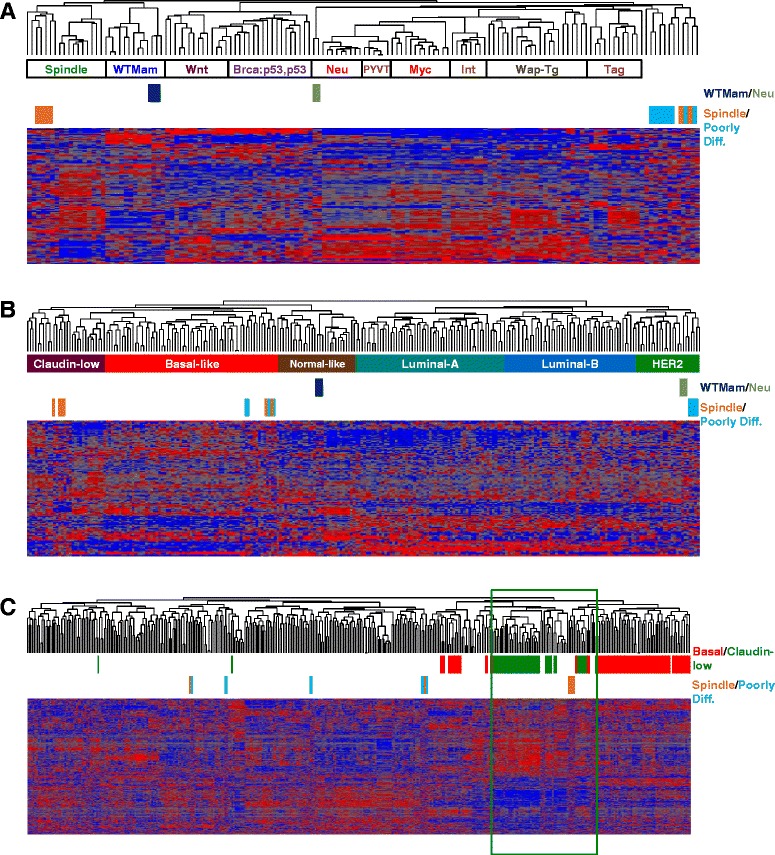
Molecular classification of PD and spindle WAP-Cre:Pten^fl/fl^:p53^R270H/wt^ mammary tumors. **a** Cluster analysis of spindle and PD WAP-Cre:Pten^fl/fl^:p53^R270H/wt^ mammary tumors using an intrinsic gene signature in comparison with 13 other mouse models of breast cancer. **b** Cluster analysis of spindle and PDAs WAP-Cre:Pten^fl/fl^:p53^R270H/wt^ mammary tumors using an intrinsic gene signature in comparison with human tumor samples. **c** Cluster analysis of WAP-Cre:Pten^fl/fl^:p53^R270H/wt^ mammary tumors with human claudin-low (*green*) and basal-like (*basal*) BC using the Prat et al. claudin-low signature. *BC* breast cancer, *PDA* poorly differentiated adenocarcinoma

To molecularly classify the Pten^∆f^:p53^R270H^ spindle and PDA tumors, we performed unsupervised hierarchical clustering on six spindle and eight PDA tumors using an extended intrinsic breast cancer signature [9, 20]. To compare gene expression across platforms we used the DWD algorithm [27]. We included several normal mammary glands and MMTV-Her2/Neu tumors as internal controls. Cluster analysis grouped the normal glands and tumors with published wild-type mammary gland and MMTV-Her2/Neu tumors, respectively, thereby validating our normalization procedure (Fig. 3a). Comparing our mammary tumors to 13 other mouse models of breast cancer [20], most (4/6) Pten^∆f^:p53^R270H^ spindle tumors clustered with other models of spindle tumors including certain p53-deficient, Brac1/p53-deficient and 7,12-dimethylbenzanthracene (DMBA)-induced mammary tumors (Fig. 4a). We attribute this incomplete consistency (4/6 rather than 6/6) to intra-tumor heterogeneity and the distinct biopsies taken for bioinformatics vs. histology. In contrast, all PDA (8/8) as well as two spindle-like tumors clustered most closely, albeit on a separate branch, with SV40 large T antigen-induced tumors, which were previously shown to resemble human basal-like breast cancer [20].

### Poorly differentiated and spindle-like Pten^∆f^:p53^R270H^ tumors cluster with human basal-like and claudin-low breast cancer

We next compared our Pten^∆f^:p53^R270H^ tumors with the five human molecular breast cancer subtypes: ERα^+^ (luminal A and luminal B), HER2, basal-like and claudin-low breast cancer as well as normal-like tumors [20]. Despite the difficulty in this interspecies analysis, four mouse Pten^∆f^:p53^R270H^ spindle tumors clustered with human claudin-low breast cancer, whereas two tumors clustered with BLBC. Four of the eight Pten^∆f^:p53^R270H^ PDAs clustered with human basal-like breast tumors, whereas four other PDAs clustered with HER2^+^ breast cancer (Fig. 4b). We note that in contrast to the original Herschkowitz et al. article, in which MMTV-Her2/neu mouse tumors clustered with human luminal breast cancer [20], in our classification, the Her2/Neu tumors clustered with human HER2^+^ breast cancer.

We next used a claudin-low signature developed by Prat et al. [21] and the GSE18229 dataset, to classify our Pten^∆f^:p53^R270H^ tumors with 397 human BC samples. The data was integrated by DWD and unsupervised clustering was done by complete linkage analysis. Again, the four mouse spindle tumors, but not the other (outliers) spindle or the eight PDA, clustered with human claudin-low BC (Fig. 4c). Thus, despite some variations due to difficulties with batch correction across different platforms and interspecies analysis as well as intra-tumor heterogeneity, most Pten^∆f^:p53^R270H^ spindle tumors and some of the PDAs clustered with human claudin-low or BLBC, respectively [20].

### Spindle-like Pten^∆f^:p53^R270H^ mutant and Pten^∆f^:p53^∆f^ deletion mammary tumors cluster closely together

**Fig. 5:**
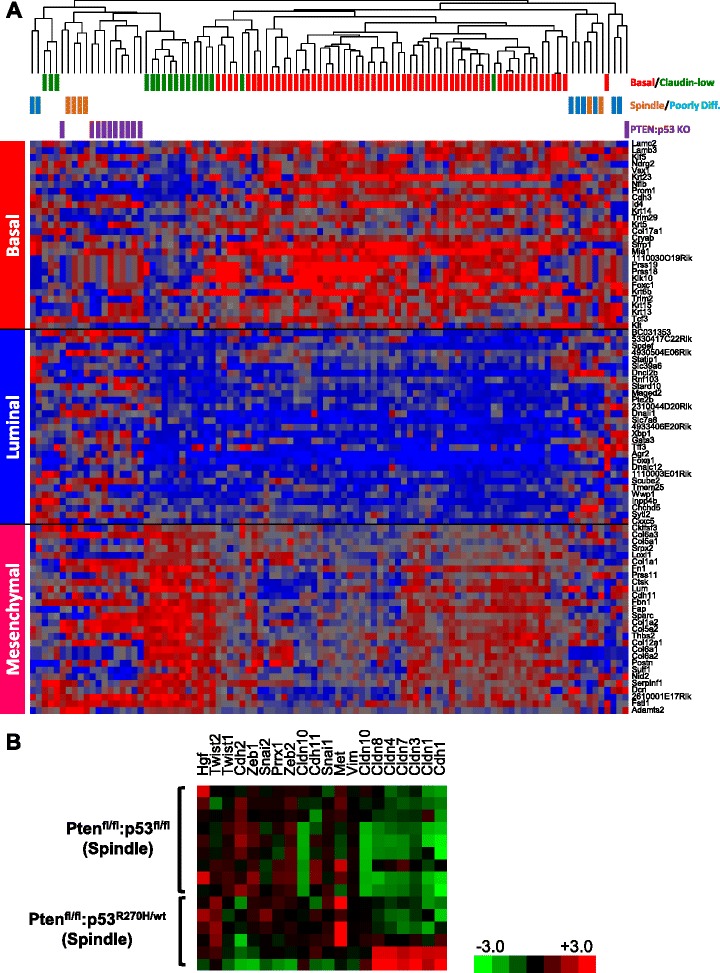
Molecular clustering of spindle-like Pten^∆f^:p53^R270H^ and Pten^∆f^:p53^∆f^ mammary tumors. **a** Hierarchical clustering using basal, luminal and mesenchymal genes showing that Pten^∆f^:p53^R270H^ and Pten^∆f^:p53^∆f^ spindle tumors grouped together with human claudin-low breast cancer, whereas Pten^∆f^:p53^R270H^ PDA and two of the spindle tumors grouped with human basal-like breast cancer. **b** Heat map showing expression of EMT genes in spindle-like Pten^∆f^:p53^R270H^ and Pten^∆f^:p53^∆f^ tumors. *EMT* epithelial to mesenchymal transition, *PDA* poorly differentiated adenocarcinoma

We directly compared the expression levels of basal, luminal and mesenchymal markers, as defined by Herschkowitz et al., [20] in WAP-Cre:Pten^f/f^:p53^lsl_R270H/+^ mutant spindle and PDA tumors as well as WAP-Cre:Pten^f/f^:p53^f/f^ double-deletion spindle tumors [9]. Classification analysis with these markers grouped the four Pten^∆f^:p53^R270H^ spindle and nearly all (10/11) Pten^∆f^:p53^∆f^ spindle tumors together with human claudin-low BC, whereas six of eight Pten^∆f^:p53^R270H^ PDA grouped together with human BLBC (Fig. 5a).

Heat map showing expression of claudins, mesenchymal and epithelial to mesenchymal transition (EMT) markers in Pten^∆f^:p53^R270H^ and Pten^∆f^:p53^∆f^ spindle tumors is shown (Fig. 5b). With the exception of the two Pten^∆f^:p53^R270H^ outliers, all tumors exhibited low expression of claudins (claudin 1, 3, 7) and high expression of EMT markers such as Twist2 and Zeb2. In contrast, N-cadherin (cdh2) was higher in Pten^∆f^:p53^∆f^ double-deletion compared with Pten^∆f^:p53^R270H^ mutant, whereas Met gene expression was relatively higher in mutant cells, suggesting that these tumors are not equally committed to EMT (see below).

### FDA-approved drug screens reveal similar sensitivity of Pten^∆f^:p53^R270H^ and Pten^∆f^:p53^∆f^ claudin-low-like tumors to 8-azaguanine but no differential sensitivity to other drugs

**Fig. 6:**
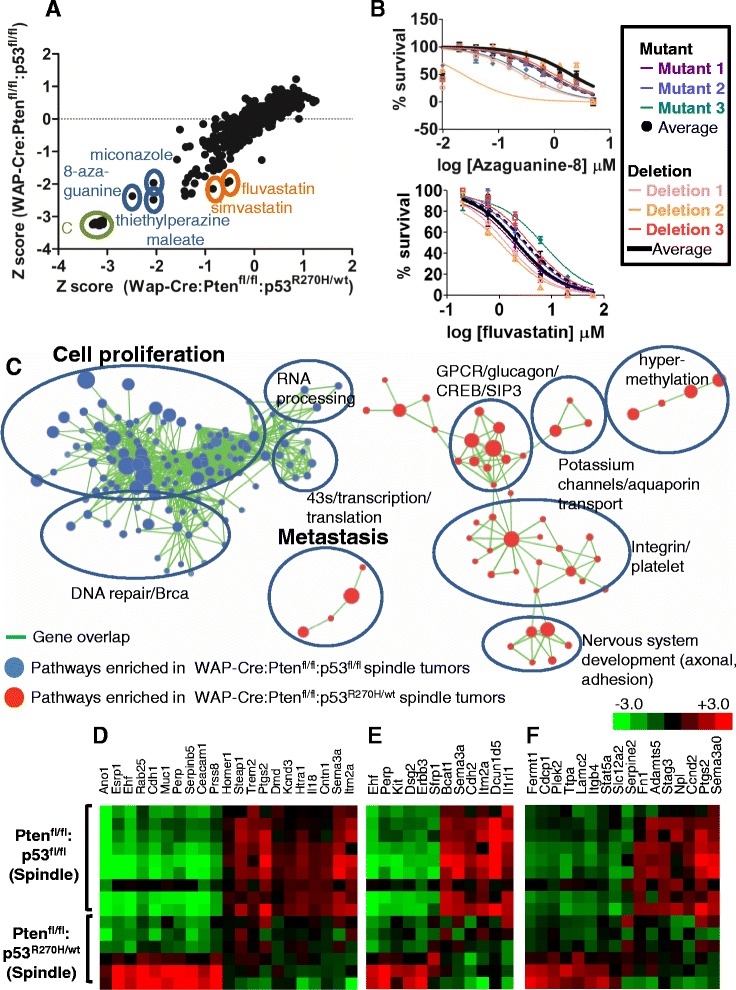
HTP screens and GSEA reveal similarities and differences between Pten^∆f^:p53^R270H^ and Pten^∆f^:p53^∆f^ mammary tumors. **a** Average response of three independent primary tumor lines from WAP-Cre:Pten^fl/fl^:p53^R270H/wt^ mice vs. three independent primary tumor lines from WAP-Cre:Pten^fl/fl^:p53^fl/fl^ mice to 1120 FDA-approved small molecules. *Highlighted* are C (no cells negative control), 8-azaguanine, miconazole, thiethylperazine malate, fluvastatin and simvastatin. **b** Dose–response curves for 8-azaguanine (average IC_50_ = 0.53 μM; *top*) and fluvastatin (IC_50_ = 0.52 μM for mutant lines; IC_50_ = 0.34 μM for deletion lines; *bottom*) using MTT viability assay. **c** GSEA showing selected pathways enriched in claudin-low-like WAP-Cre:Pten^fl/fl^:p53^R270H/wt^ (*red*) versus pathways enriched in WAP-Cre:Pten^fl/fl^:p53^wt/wt^ (*blue*) tumors. *Green lines* connect overlapping pathways. Proliferation and metastasis pathways are *highlighted*. **d**-**f** Heat map showing expression of metastasis (**d**), proliferation (**e**) and DNA repair (**d**) genes in Pten^∆f^:p53^R270H^ vs. Pten^∆f^:p53^∆f^ spindle-like tumors. *HTP* high-throughput, *GSEA*, gene set enrichment analysis

In TNBC, p53 is disrupted by deletions or by mutations in the DBD [4]. Identification of drugs that can efficiently kill both tumor types or selectively target p53-deletion vs. p53-mutant-driven breast cancer are therefore of great interest. To identify such drugs, we set up a repurposing screen to identify FDA-approved drugs with common or differential effects on Pten^∆f^:p53^R270H^ vs. Pten^∆f^:p53^∆f^ tumors. We established and compared three spindle-like primary Pten^∆f^:p53^R270H^ and three Pten^∆f^:p53^∆f^ tumor cells, using HTP robotic screens of 1120 FDA-approved drugs (Prestwick library). The top three drugs that most efficiently suppressed all six tumor lines were the purine analog, 8-azaguanine, the imidazole antifungal agent, miconazole, and the dopamine antagonist, thiethylperazine malate (Fig. 6a). Despite growing interest in the effect of dopamine antagonists on cancer stem cells [28], we suspect that the effects of miconazole and thiethylperazine are not specific because multiple other imidazoles and dopamine inhibitors present in our FDA-approved library had much lower, often agonistic effects on tumor growth (Additional file 2; Discussion). Sensitivity of Pten^∆f^:p53^∆f^ and Pten^∆f^:p53^R270H^ tumor cells to 8-azaguanine was validated on the six lines revealing IC_50_ of 0.53 μM (Fig. 6b top; Discussion).

Tumors driven by a p53 DBD mutation are addicted to its continuous expression [29–31]. Addiction is at least in part due to mutant p53-mediated transcriptional activation of the mevalonate pathway, leading to increased sensitivity to statins, which inhibit 3-hydroxy-3-methyl-glutaryl-CoA (HMG-CoA) reductase, the enzyme that produces mevalonate [31]. Importantly, depletion of mutant p53 in these tumors leads to cell demise. This was observed by knocking down p53 mutant expression via RNA interference or through HSP90 inhibitors, which destabilize mutant p53. Accordingly, we found that knocking down p53^R270H^ protein expression via lenti-shRNA significantly reduced growth of primary Pten^∆f^:p53^R270H^ tumor cells (data not shown). However, selective inhibition of p53-mutant vs. p53-deleted TNBC may be achieved not only by destabilizing mutant p53 but also by targeting distinct oncogenic genes/pathways that cooperate with each p53 allele (i.e. mutation vs. deletion). We therefore asked whether any of the 1120 FDA-approved drugs had differential inhibitory effect on Pten^∆f^:p53^∆f^ vs. Pten^∆f^:p53^R270H^ tumor cells. Surprisingly, our screens identified two statins (fluvastatin and simvastatin) (Fig. 6a), as well as four other drugs (thiostrepton, podophyllotoxin, parbendazole, mebendazole) as having differential effects on Pten^∆f^:p53^R270H^ vs. Pten^∆f^:p53^∆f^. Upon robotic re-screens, Pten^∆f^:p53^∆f^ showed increased sensitivity relative to Pten^∆f^:p53^R270H^ tumor cells to low concentrations of fluvastatin and simvastatin but not to the other drugs (Additional files 3 and 4). This mild increase in sensitivity to statins was not reproducible or robust enough to show significance in subsequent validation experiments (*p* = 0.3341) (Fig. 6b; *bottom*). Taken together, using highly similar Pten^∆f^:p53^∆f^ vs. Pten^∆f^:p53^R270H^ claudin-low like tumor cells we found no robust differences in sensitivity to 1120 FDA-approved drugs (see Discussion).

### Claudin-low-like and basal-like Pten^∆f^:p53^R270H^ tumor cells have higher metastatic potential than Pten^∆f^:p53^∆f^ spindle mammary tumor cells

Results from our cluster analysis and HTP screens suggested that despite the similarity, Pten^∆f^:p53^R270H^ and Pten^∆f^:p53^∆f^ spindle tumors exhibited variances. To explore such differences further, we performed pathway analysis using GSEA. Pathways enriched in the Pten^∆f^:p53^∆f^ spindle tumors (*blue* in Fig. 6c) were associated with cell proliferation and DNA repair/Brca, whereas pathways induced in Pten^∆f^:p53^R270H^ spindle tumors (*red*) were related to metastasis and other pathways (heat map of selected genes Fig. 6d-f).

**Fig. 7:**
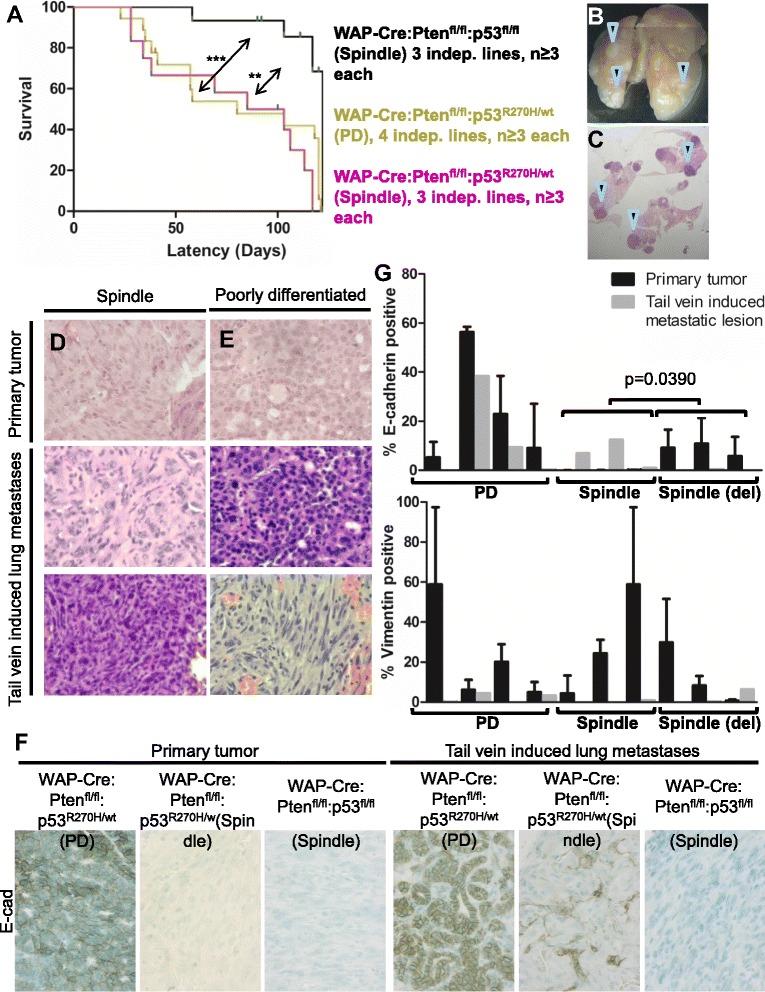
WAP-Cre:Pten^fl/fl^:p53^R270H/wt^ spindle and PDA tumor cells exhibit higher metastatic potential than WAP-Cre:Pten^fl/fl^:p53^fl/fl^ spindle tumor cells. **a** Kaplan-Meier survival curve of NOD/SCID female mice following tail vein injection with indicated primary tumor cells showing that p53^R270H^ mutation accelerated metastatic formation relative to p53 deletion. Mice were euthanized when moribund. Statistical significance by Wilcoxon method: spindle Pten^fl/fl^/p53^fl/fl^ vs. spindle Pten^fl/fl^/p53^R270H^, *p* = 0.0004; spindle Pten^fl/fl^/p53^fl/fl^ vs. PDA Pten^fl/fl^/p53^R270H^, *p* = 0.0012; spindle Pten^fl/fl^/p53^R270H^ vs. PDA Pten^fl/fl^/p53^R270H^, *p* = 0.16. **b**-**e**. Representative whole lung (**b**) and H&E images (**c**) of mice injected with WAP- Cre:Pten^fl/fl^:p53^R270H/wt^ mammary tumor cells at end point. *Arrowheads* indicate metastatic lesions. Representative H&E images of (**d**) primary spindle and (**e**) primary PDA WAP-Cre:Pten^fl/fl^:p53^R270H/wt^ mammary tumors and their metastatic counterparts. Note that one of the metastatic lesions from the PDA had spindle-shaped morphology (*bottom right*). **f** Representative images of E-cadherin expression in primary WAP-Cre:Pten^fl/fl^:p53^R270H/wt^ and WAP-Cre:Pten^fl/fl^:p53^fl/fl^ mammary tumors and lung metastases. **g** Expression levels of epithelial (E-cadherin) and mesenchymal (vimentin) markers in primary WAP-Cre:Pten^fl/fl^:p53^R270H/wt^ and WAP-Cre:Pten^fl/fl^:p53^fl/fl^ mammary tumors and lung metastases. *H&E* hematoxylin and eosin, *PDA* poorly differentiated adenocarcinoma

To functionally test the latter observation, we determined the metastatic potential of primary Pten^∆f^:p53^R270H^ spindle-like and PDA relative to primary Pten^∆f^:p53^∆f^ spindle tumor cells using tail vein injection assays. Although this assay does not monitor invasion and intravasation through the basal membrane into a blood or lymphatic vessel – it does measure for the ability of tumor cells to survive in the circulating system, extravasate and form macrometastases in distal sites, a rate-limiting step in the metastatic cascade. Following engraftment, mice were closely monitored for breathing difficulty, weight loss and sickness. Using these criteria, end points following transplantation of Pten^∆f^:p53^R270H^ spindle and PDA tumor cells were significantly shorter than for Pten^∆f^:p53^∆f^ double-deletion tumors (Fig. 7a). Upon autopsy, lungs from all engrafted mice contained macroscopic nodules, which were confirmed as lung metastases by histological examination (Fig. 7b-c). Like the TIC assay (Fig. 3), the lung metastases from spindle tumors invariable shared the same spindle-shaped morphology as the parental tumors from which they were derived, whereas primary PDAs gave rise to metastases that showed either PDA or spindle morphology (Fig. 7d-e). These observations point to subclonal diversity/intra-tumor heterogeneity and more efficient metastasis by Pten^∆f^:p53^R270H^ spindle tumor cells.

Primary Pten^∆f^:p53^∆f^ spindle tumors and their metastases showed near complete absence of the epithelial marker E-cadherin. In contrast, while Pten^∆f^:p53^R270H^ primary spindle tumors were largely negative for E-cadherin, lung metastases expressed significantly more of this transmembrane glycoprotein than primary lesions; PDA primary tumors were positive for E-cadherin to varying degrees, and this was largely preserved in metastatic counterparts (Fig. 7f-g). While E-cadherin expression increased in Pten^∆f^:p53^R270H^ metastases, expression of the mesenchymal marker vimentin was reduced (Fig. 7g, Additional file 5). Together these results suggest that increased metastatic potential of Pten^∆f^:p53^R270H^ relative to Pten^∆f^:p53^∆f^ tumors may be attributed at least in part to their ability to undergo partial mesenchymal to epithelial (MET) conversion, which is required for efficient colonization and growth at distal sites [32].

## Discussion

We show that targeted deletion of the tumor suppressor Pten together with expression of a p53 DBD mutant, R270H, in mammary epithelium via WAP-Cre induces diverse mammary tumor subtypes including PDA and spindle tumors. Intra- and inter-species cluster analysis classified most PDA and spindle tumors as basal-like and claudin-low like TNBC, respectively. In stark contrast, double-deletion tumors from WAP-Cre:Pten^f/f^:p53^f/f^ mice invariably cluster with human claudin-low breast cancer [9]. Claudin-low-like TNBC were also found when Pten and p53, or Rb plus p53 were homozygously deleted via MMTV-Cre [9, 17], or when the MET oncogene was overexpressed in mammary epithelium together with homozygous p53 deletion [33]. Indeed, these articles showed that homozygous loss of p53 alone induces many, though not exclusively, claudin-low-like tumors, indicating that deletion of this tumor suppressor in the mouse directs tumorigenesis toward this subtype. The Pten^∆f^:p53^R270H^ PDAs expressed the basal keratins K5 and K6, clustered with mouse models of BLBC and with human BLBC samples, exhibited high TIC frequency, and readily formed lung metastases following tail vein injections. Thus, WAP-Cre:Pten^f/f^:p53^lsl_R270H/+^ mice offer a new model to study basal-like as well as claudin-low Pten/p53-deficient TNBC. Our results also suggest that WAP-Cre:p53^lsl_R270H/+^ rather than WAP-Cre:p53^f/f^ mice should be used to model human breast cancer in conjunction with mutations in other breast cancer drivers.

Pten^∆f^:p53^∆f^ and Pten^∆f^:p53^R270H^ tumors provide the means to compare histologically indistinguishable tumors with either mutation of deletion of p53. We therefore performed FDA-approved drug screens to identify compounds that can kill both tumors or preferentially target tumors driven by p53 deletion or mutation. We found that 8-azaguanine, a purine analog, efficiently killed both Pten^∆f^:p53^∆f^ and Pten^∆f^:p53^R270H^ tumors. Sensitivity to 8-azaguanine may reflect high cell proliferation or low levels of guanine deaminase, which can convert this purine analog to a noncytotoxic metabolite (8-azaxanthine) [34]. 8-azaguanine incorporation via hypoxanthine guanine phosphoribosyl transferase (HGPRTase) leads to inhibition of purine nucleotide synthesis. It may also be toxic due to its incorporation into RNA [35]. Despite some benefits to leukemia patients, adverse dermatological reactions, nausea and vomiting limit its use [36]. Additional analysis is required to assess possible benefits, if any, of 8-azaguanine either alone or in combination with PI3K antagonists or other inhibitors for Pten/p53-deficient TNBC. Notably, in a kinome screen of Pten^∆f^:p53^∆f^ tumors we identified eEF2K antagonists as better inhibitors than AKT and PI3K inhibitors [9]. Such pathway-specific drugs are much more likely to have a large therapeutic window as compared to cytotoxic drugs like 8-azaguanine. Additional screens with both Pten^∆f^:p53^∆f^ and Pten^∆f^:p53^R270H^ tumors as well as with human Pten/p53-deficient breast cancer lines using large libraries of divergent compounds may uncover new potent drugs for this aggressive cancer subtype.

Contrary to published results on increased sensitivity of mutant p53 to statins [31], we observed consistent but mild and statistically insignificant increase in sensitivity of Pten^∆f^:p53^∆f^ tumor cells to HMG-CoA inhibitors relative to Pten^∆f^:p53^R270H^ tumor cells. One explanation for this discrepancy may be that although p53 mutation alone increases sensitivity to statins by inducing the mevalonate pathway, tumors that develop in the absence of p53 (p53 deletion) may increase sensitivity to statins through other mechanisms. Indeed, we found that a signature that predicts sensitivity to statins [37] is elevated in Pten^∆f^:p53^∆f^ relative to Pten^∆f^:p53^R270H^ tumor cells (not shown). We note that the FDA-approved drug library we used here does not include the HSP90 inhibitor geldanamycin or its more potent derivatives, which destabilize mutant p53. However, we found that knockdown of p53^R270H^ protein impeded cell growth (not shown) indicating that Pten^∆f^:p53^R270H^ tumor cells are addicted to continuous expression of mutant p53.

Despite – and perhaps because of – the inter- and intra-tumor heterogeneity seen in WAP-Cre:Pten^f/f^:p53^lsl_R270H/+^ mice, they offer certain advantages over WAP-Cre:Pten^f/f^:p53^f/f^ mice. First, they develop PDA/basal-like tumors in approximately 10 % of mice and spindle/claudin-low-like tumors in approximately 30 % of mice. Tumor-bearing animals can be biopsied to determine histology and then treated with candidate drugs, allowing their effect to be tested on diverse tumor types with similar oncogenic initiation events (Pten loss; p53 mutation). Alternatively, as described herein, tumors can be harvested, frozen, classified and then transplanted to obtain multiple xenografts with uniform tumor subtype. Together, the WAP-Cre:Pten^f/f^:p53^lsl_R270H/+^ and WAP-Cre:Pten^f/f^:p53^f/f^ mice model the spectrum of p53 gene aberrations and Pten loss seen in human TNBC.

Using GSEA and functional analysis we showed that Pten^∆f^:p53^R270H^ tumor cells are more metastatic than Pten^∆f^:p53^∆f^ tumors and this was correlated with induction of E-cadherin in lung metastases from Pten^∆f^:p53^R270H^ tumor cells. P53 mutations in the DBD were shown to increase metastasis through induction of EMT via several routes including sequestration of p63, stabilization/activation of SLUG/SNAIL, induction of the mir130b-Zeb1 axis, TWIST and SLUG transcription factors and other mechanisms [12, 38–42]. Our study suggests that once disseminating mutant p53 tumors home at distal sites, they more readily undergo MET, which enhances colonization, the dominant rate-limiting step of the metastatic cascade [32, 43, 44].

## Conclusions

WAP-Cre:Pten^f/f^:p53^lox.stop.lox_R270H/+^ mice described herein together with WAP-Cre:Pten^f/f^:p53^f/f^ mice [9], provide powerful preclinical models to interrogate and decipher the genetic cooperation that drive primary basal-like and claudin-low TNBC development and metastatic spread/colonization, as well as identify and test for novel therapeutics that target these aggressive breast cancers.

## Additional files

Additional file 1:
**Representative large images of histology of primary WAP-Cre:Pten** ^**fl/fl**^ **:p53** ^**R270H/wt**^ **mammary tumors shown in Fig.** 1 **.** (PPT 1510 kb) Additional file 2:
**Z scores for response to dopamine antagonist (amoxapine, clomipramine hydrochloride, clozapine, domperidone maleate, droperidol, metoclopramide monohydrochloride, risperidone, thiethylperazine malate, thioridazine hydrochloride, trifluoperazine dihydrochloride, and trimipramine maleate salt) and imidazole antifungal (butoconazole nitrate, clotrimazole, econazole nitrate, isoconazole, ketoconazole, miconazole, sertaconazole nitrate, and sulconazole nitrate) treatment of three independent primary WAP-Cre:Pten** ^**fl/fl**^ **:p53** ^**R270H/wt**^ **tumor lines and three independent primary WAP-Cre:Pten** ^**fl/fl**^ **:p53** ^**fl/fl**^ **tumor lines.** (PPT 102 kb) Additional file 3:
**Dose–response curves for fluvastatin and simvastatin in three independent primary WAP-Cre:Pten** ^**fl/fl**^ **:p53** ^**R270H/wt**^ **tumor lines and three independent primary WAP-Cre:Pten** ^**fl/fl**^ **:p53** ^**fl/fl**^ **tumor lines.** Insignificant *P* values for both curves. In addition, for simvastatin, when the six lines were analyzed independently, IC_50_ for the three mutant lines were: 2.829, 2.474 and 0.1096; IC_50_ for the three deletion lines were: 0.01233, 0.1911 and 0.01995. *P* value of these six IC_50_ values was 0.18. (PPT 108 kb) Additional file 4:
**Dose–response curves for thiostrepton, podophyllotoxin, mebendazole and parbendazole in three independent primary WAP-Cre:Pten** ^**fl/fl**^ **:p53** ^**R270H/wt**^ **tumor lines and three independent primary WAP-Cre:Pten** ^**fl/fl**^ **:p53** ^**fl/fl**^ **tumor lines.** Insignificant *P* values for all curves. (PPT 930 kb) Additional file 5:
**Representative images of vimentin immunostaining of primary WAP-Cre:Pten** ^**fl/fl**^ **:p53** ^**R270H/wt**^ **and WAP-Cre:Pten** ^**fl/fl**^ **:p53** ^**fl/fl**^ **mammary tumors and lung metastases (quantification in Fig.** 7 **).** (PPT 930 kb)

## Abbreviations

AME: Adenomyoepithelioma
BC: Breast cancer
BLBC: basal-like breast cancer
DBD: DNA-binding domain
DMEM: Dulbecco’s modified Eagle’s medium
DWD: Distance-weighted discrimination
EGF: Epidermal growth factor
EMT: Epithelial to mesenchymal transition
ERα: Estrogen receptor alpha
FDA: Food and Drug Administration
FGF: Fibroblast growth factor
GSEA: Gene set enrichment analysis
HER2: Human epidermal growth factor receptor 2
HMG-CoA: 3-hydroxy-3- methyl-glutaryl-CoA
HTP: High-throughput
IHC: Immunohistochemistry
MET: Mesenchymal to epithelial transition
MMTV: Mouse mammary tumor virus
PCR: Polymerase chain reaction
PDA: Poorly differentiated adenocarcinoma
PIP2: Phosphatidylinositol (4, 5)-disphosphate
PIP3: Phosphatidylinositol (3, 4, 5)-trisphosphate
PTEN: Phosphatase and tensin homolog deleted in chromosome 10
TIC: Tumor-initiating cell
TNBC: Triple-negative breast cancer
WAP: Whey acidic protein

## References

[1] Prat A, Adamo B, Cheang MC, Anders CK, Carey LA, Perou CM (2013). Molecular characterization of basal-like and non-basal-like triple-negative breast cancer. Oncologist..

[2] Curtis C, Shah SP, Chin SF, Turashvili G, Rueda OM, Dunning MJ (2012). The genomic and transcriptomic architecture of 2,000 breast tumours reveals novel subgroups. Nature..

[3] Banerji S, Cibulskis K, Rangel-Escareno C, Brown KK, Carter SL, Frederick AM (2012). Sequence analysis of mutations and translocations across breast cancer subtypes. Nature..

[4] Cancer Genome Atlas Network (2012). Comprehensive molecular portraits of human breast tumours. Nature..

[5] Lehmann BD, Bauer JA, Chen X, Sanders ME, Chakravarthy AB, Shyr Y (2011). Identification of human triple-negative breast cancer subtypes and preclinical models for selection of targeted therapies. J Clin Invest..

[6] Cully M, You H, Levine AJ, Mak TW (2006). Beyond PTEN mutations: the PI3K pathway as an integrator of multiple inputs during tumorigenesis. Nat Rev Cancer..

[7] Hopkins BD, Hodakoski C, Barrows D, Mense SM, Parsons RE (2014). PTEN function: the long and the short of it. Trends Biochem Sci..

[8] Stambolic V (2015). Cancer: Precise control of localized signals. Nature..

[9] Liu JC, Voisin V, Wang S, Wang DY, Jones RA, Datti A (2014). Combined deletion of Pten and p53 in mammary epithelium accelerates triple-negative breast cancer with dependency on eEF2K. EMBO Mol Med..

[10] Herschkowitz JI, Zhao W, Zhang M, Usary J, Murrow G, Edwards D (2012). Comparative oncogenomics identifies breast tumors enriched in functional tumor-initiating cells. Proc Natl Acad Sci U S A..

[11] Olive KP, Tuveson DA, Ruhe ZC, Yin B, Willis NA, Bronson RT (2004). Mutant p53 gain of function in two mouse models of Li-Fraumeni syndrome. Cell..

[12] Powell E, Piwnica-Worms D, Piwnica-Worms H (2014). Contribution of p53 to metastasis. Cancer Discov..

[13] Wijnhoven SW, Speksnijder EN, Liu X, Zwart E, vanOostrom CT, Beems RB (2007). Dominant-negative but not gain-of-function effects of a p53.R270H mutation in mouse epithelium tissue after DNA damage. Cancer Res.

[14] Wagner KU, Wall RJ, St-Onge L, Gruss P, Wynshaw-Boris A, Garrett L (1997). Cre-mediated gene deletion in the mammary gland. Nucleic Acids Res..

[15] Suzuki A, de la Pompa JL, Stambolic V, Elia AJ, Sasaki T, del Barco BI (1998). High cancer susceptibility and embryonic lethality associated with mutation of the PTEN tumor suppressor gene in mice. Curr Biol..

[16] Wagner KU, Boulanger CA, Henry MD, Sgagias M, Hennighausen L, Smith GH (2002). An adjunct mammary epithelial cell population in parous females: its role in functional adaptation and tissue renewal. Development..

[17] Jiang Z, Deng T, Jones R, Li H, Herschkowitz JI, Liu JC (2010). Rb deletion in mouse mammary progenitors induces luminal-B or basal-like/EMT tumor subtypes depending on p53 status. J Clin Invest..

[18] Deng T, Liu JC, Chung PE, Uehling D, Aman A, Joseph B (2014). shRNA kinome screen identifies TBK1 as a therapeutic target for HER2+ breast cancer. Cancer Res.

[19] Liu JC, Voisin V, Bader GD, Deng T, Pusztai L, Symmans WF (2012). Seventeen-gene signature from enriched Her2/Neu mammary tumor-initiating cells predicts clinical outcome for human HER2+:ERalpha- breast cancer. Proc Natl Acad Sci U S A..

[20] Herschkowitz JI, Simin K, Weigman VJ, Mikaelian I, Usary J, Hu Z (2007). Identification of conserved gene expression features between murine mammary carcinoma models and human breast tumors. Genome Biol..

[21] Prat A, Parker JS, Karginova O, Fan C, Livasy C, Herschkowitz JI (2010). Phenotypic and molecular characterization of the claudin-low intrinsic subtype of breast cancer. Breast Cancer Res..

[22] Subramanian A, Tamayo P, Mootha VK, Mukherjee S, Ebert BL, Gillette MA (2005). Gene set enrichment analysis: a knowledge-based approach for interpreting genome-wide expression profiles. Proc Natl Acad Sci U S A..

[23] Merico D, Isserlin R, Stueker O, Emili A, Bader GD (2011). Enrichment map: a network-based method for gene-set enrichment visualization and interpretation. PLoS One..

[24] Brideau C, Gunter B, Pikounis B, Liaw A (2003). Improved statistical methods for hit selection in high-throughput screening. J Biomol Screen..

[25] Magee JA, Piskounova E, Morrison SJ (2012). Cancer stem cells: impact, heterogeneity, and uncertainty. Cancer Cell..

[26] Kreso A, Dick JE (2014). Evolution of the cancer stem cell model. Cell Stem Cell..

[27] Benito M, Parker J, Du Q, Wu J, Xiang D, Perou CM (2004). Adjustment of systematic microarray data biases. Bioinformatics..

[28] Sachlos E, Risueno RM, Laronde S, Shapovalova Z, Lee JH, Russell J (2012). Identification of drugs including a dopamine receptor antagonist that selectively target cancer stem cells. Cell..

[29] Weissmueller S, Manchado E, Saborowski M, Morris JP, Wagenblast E, Davis CA (2014). Mutant p53 drives pancreatic cancer metastasis through cell-autonomous PDGF receptor beta signaling. Cell..

[30] Alexandrova EM, Yallowitz AR, Li D, Xu S, Schulz R, Proia DA (2015). Improving survival by exploiting tumour dependence on stabilized mutant p53 for treatment. Nature..

[31] Freed-Pastor WA, Mizuno H, Zhao X, Langerod A, Moon SH, Rodriguez-Barrueco R (2012). Mutant p53 disrupts mammary tissue architecture via the mevalonate pathway. Cell..

[32] Brabletz T (2012). EMT and MET in metastasis: where are the cancer stem cells?. Cancer Cell..

[33] Knight JF, Lesurf R, Zhao H, Pinnaduwage D, Davis RR, Saleh SM (2013). Met synergizes with p53 loss to induce mammary tumors that possess features of claudin-low breast cancer. Proc Natl Acad Sci U S A..

[34] Meyers MB, Shin S (1981). Specific resistance to 8-azaguanine in cells with normal hypoxanthine phosphoribosyltransferase (HPRT) activity: the role of guanine deaminase. Cytogenet Cell Genet..

[35] Nelson JA, Carpenter JW, Rose LM, Adamson DJ (1975). Mechanisms of action of 6-thioguanine, 6-mercaptopurine, and 8-azaguanine. Cancer Res..

[36] Colsky J, Meiselas LE, Rosen SJ, Schulman I (1955). Response of patients with leukemia to 8-azaguanine. Blood..

[37] Goard CA, Chan-Seng-Yue M, Mullen PJ, Quiroga AD, Wasylishen AR, Clendening JW (2014). Identifying molecular features that distinguish fluvastatin-sensitive breast tumor cells. Breast Cancer Res Treat..

[38] Adorno M, Cordenonsi M, Montagner M, Dupont S, Wong C, Hann B (2009). A mutant-p53/Smad complex opposes p63 to empower TGFbeta-induced metastasis. Cell..

[39] Senoo M, Pinto F, Crum CP, McKeon F (2007). p63 is essential for the proliferative potential of stem cells in stratified epithelia. Cell..

[40] Wang SP, Wang WL, Chang YL, Wu CT, Chao YC, Kao SH (2009). p53 controls cancer cell invasion by inducing the MDM2-mediated degradation of Slug. Nat Cell Biol..

[41] Godar S, Ince TA, Bell GW, Feldser D, Donaher JL, Bergh J (2008). Growth-inhibitory and tumor- suppressive functions of p53 depend on its repression of CD44 expression. Cell..

[42] Muller PA, Caswell PT, Doyle B, Iwanicki MP, Tan EH, Karim S (2009). Mutant p53 drives invasion by promoting integrin recycling. Cell..

[43] Ocana OH, Corcoles R, Fabra A, Moreno-Bueno G, Acloque H, Vega S (2012). Metastatic colonization requires the repression of the epithelial-mesenchymal transition inducer Prrx1. Cancer Cell..

[44] Tsai JH, Donaher JL, Murphy DA, Chau S, Yang J (2012). Spatiotemporal regulation of epithelial-mesenchymal transition is essential for squamous cell carcinoma metastasis. Cancer Cell..

