# Supplementary figures and images for: Targeted Pten deletion plus p53-R270H mutation in mouse mammary epithelium induces aggressive claudin-low and basal-like breast cancer
Source: Breast Cancer Res. 2016 Jan 19;18:9. doi: 10.1186/s13058-015-0668-y (PMC4717616; doi:10.1186/s13058-015-0668-y)

## Slide 1
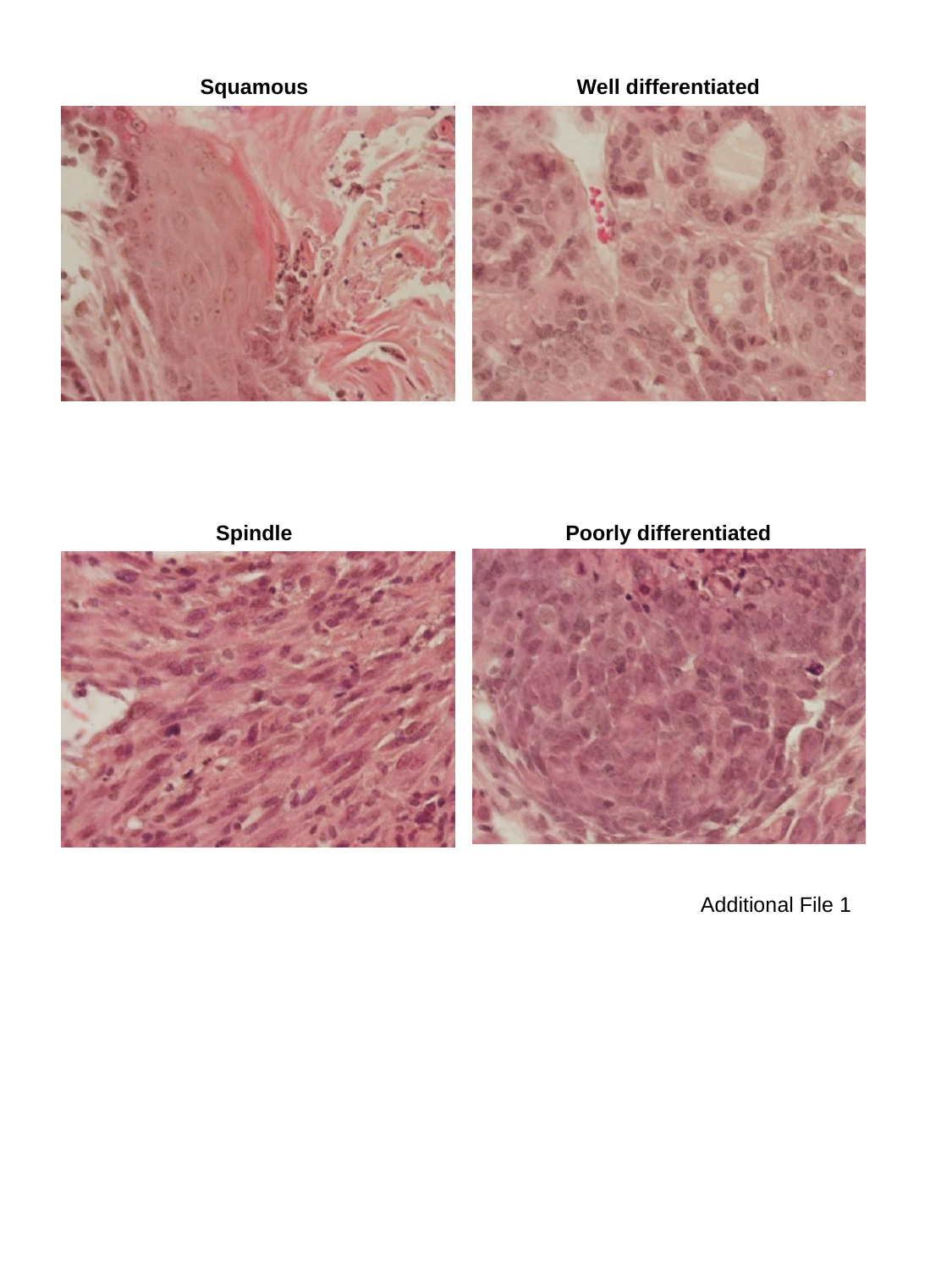

Squamous
Well differentiated
Spindle
Poorly differentiated
Additional File 1

Supplement: Additional file 1: — Representative large images of histology of primary WAP-Cre:Pten fl/fl :p53 R270H/wt mammary tumors shown in Fig. 1 . (PPT 1510 kb) [file 13058_2015_668_MOESM1_ESM.ppt]

## Slide 1
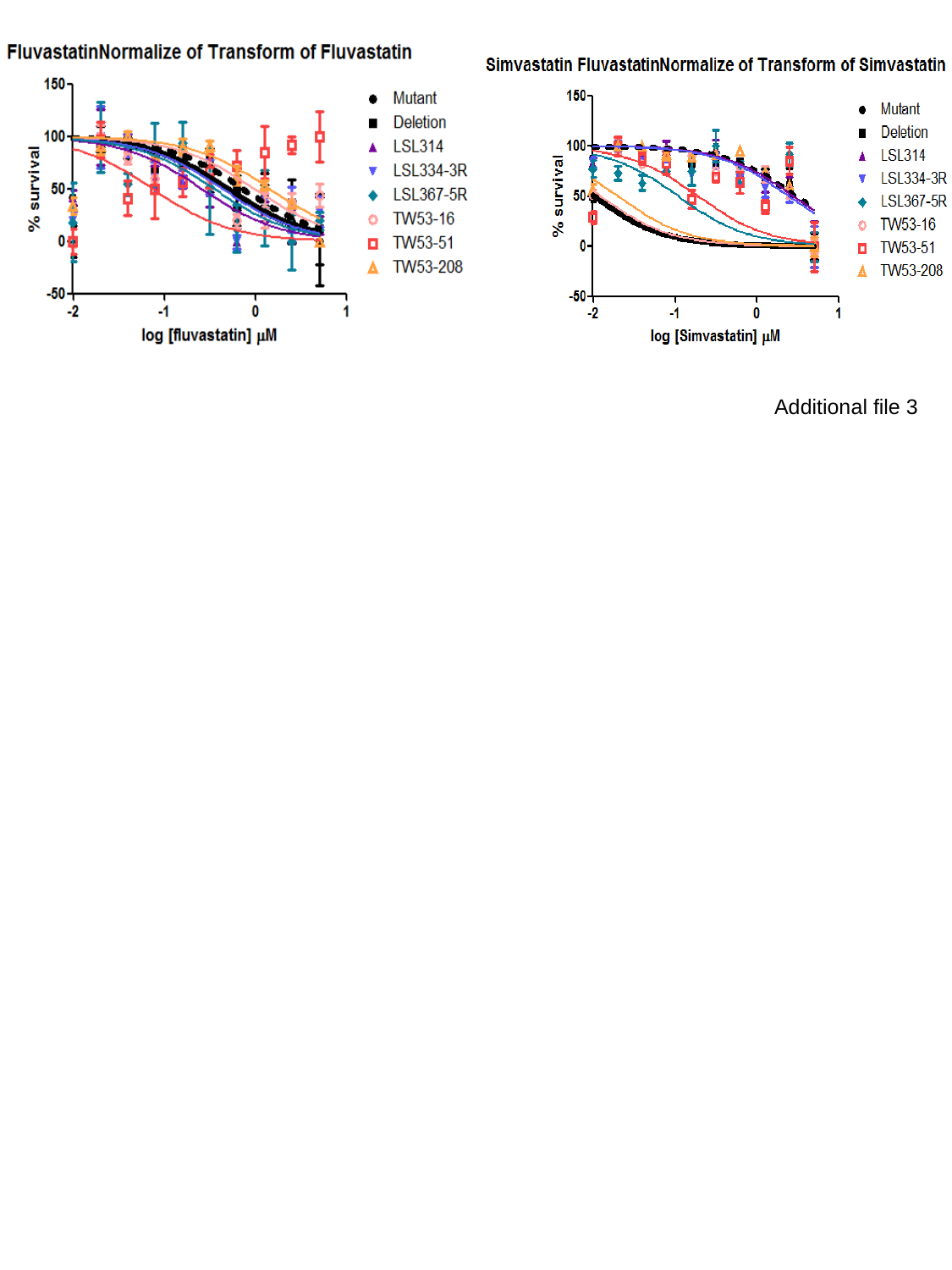

# Additional file 3

Supplement: Additional file 3: — Dose–response curves for fluvastatin and simvastatin in three independent primary WAP-Cre:Pten fl/fl :p53 R270H/wt tumor lines and three independent primary WAP-Cre:Pten fl/fl :p53 fl/fl tumor lines. Insignificant P values for both curves. In addition, for simvastatin, when the six lines were analyzed independently, IC50 for the three mutant lines were: 2.829, 2.474 and 0.1096; IC50 for the three deletion lines were: 0.01233, 0.1911 and 0.01995. P value of these six IC50 values was 0.18. (PPT 108 kb) [file 13058_2015_668_MOESM3_ESM.ppt]
