# Supplementary material for: Targeted Pten deletion plus p53-R270H mutation in mouse mammary epithelium induces aggressive claudin-low and basal-like breast cancer
Source: Breast Cancer Res. 2016 Jan 19;18:9. doi: 10.1186/s13058-015-0668-y (PMC4717616; doi:10.1186/s13058-015-0668-y)
Supplement: Additional file 2: — Z scores for response to dopamine antagonist (amoxapine, clomipramine hydrochloride, clozapine, domperidone maleate, droperidol, metoclopramide monohydrochloride, risperidone, thiethylperazine malate, thioridazine hydrochloride, trifluoperazine dihydrochloride, and trimipramine maleate salt) and imidazole antifungal (butoconazole nitrate, clotrimazole, econazole nitrate, isoconazole, ketoconazole, miconazole, sertaconazole nitrate, and sulconazole nitrate) treatment of three independent primary WAP-Cre:Pten fl/fl :p53 R270H/wt tumor lines and three independent primary WAP-Cre:Pten fl/fl :p53 fl/fl tumor lines. (PPT 102 kb) [file 13058_2015_668_MOESM2_ESM.ppt]

## Slide 1
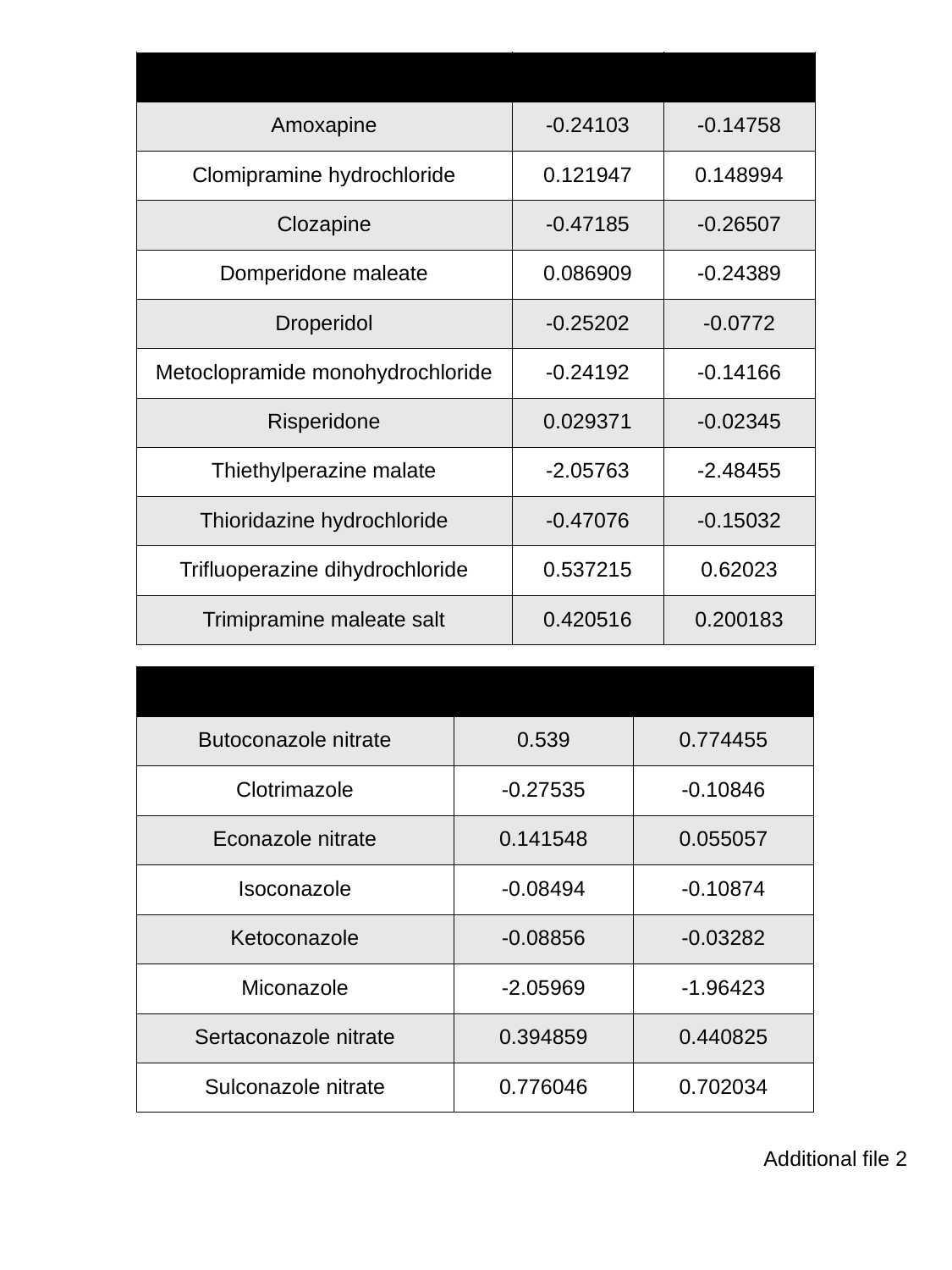

| Dopamine antagonists | Mutant | Deletion |
| --- | --- | --- |
| Amoxapine | -0.24103 | -0.14758 |
| Clomipramine hydrochloride | 0.121947 | 0.148994 |
| Clozapine | -0.47185 | -0.26507 |
| Domperidone maleate | 0.086909 | -0.24389 |
| Droperidol | -0.25202 | -0.0772 |
| Metoclopramide monohydrochloride | -0.24192 | -0.14166 |
| Risperidone | 0.029371 | -0.02345 |
| Thiethylperazine malate | -2.05763 | -2.48455 |
| Thioridazine hydrochloride | -0.47076 | -0.15032 |
| Trifluoperazine dihydrochloride | 0.537215 | 0.62023 |
| Trimipramine maleate salt | 0.420516 | 0.200183 |
| Imidazole antifungal | Mutant | Deletion |
| --- | --- | --- |
| Butoconazole nitrate | 0.539 | 0.774455 |
| Clotrimazole | -0.27535 | -0.10846 |
| Econazole nitrate | 0.141548 | 0.055057 |
| Isoconazole | -0.08494 | -0.10874 |
| Ketoconazole | -0.08856 | -0.03282 |
| Miconazole | -2.05969 | -1.96423 |
| Sertaconazole nitrate | 0.394859 | 0.440825 |
| Sulconazole nitrate | 0.776046 | 0.702034 |
# Additional file 2
