# Supplementary material for: Targeted Pten deletion plus p53-R270H mutation in mouse mammary epithelium induces aggressive claudin-low and basal-like breast cancer
Source: Breast Cancer Res. 2016 Jan 19;18:9. doi: 10.1186/s13058-015-0668-y (PMC4717616; doi:10.1186/s13058-015-0668-y)
Supplement: Additional file 4: — Dose–response curves for thiostrepton, podophyllotoxin, mebendazole and parbendazole in three independent primary WAP-Cre:Pten fl/fl :p53 R270H/wt tumor lines and three independent primary WAP-Cre:Pten fl/fl :p53 fl/fl tumor lines. Insignificant P values for all curves. (PPT 930 kb) [file 13058_2015_668_MOESM4_ESM.ppt]

## Slide 1
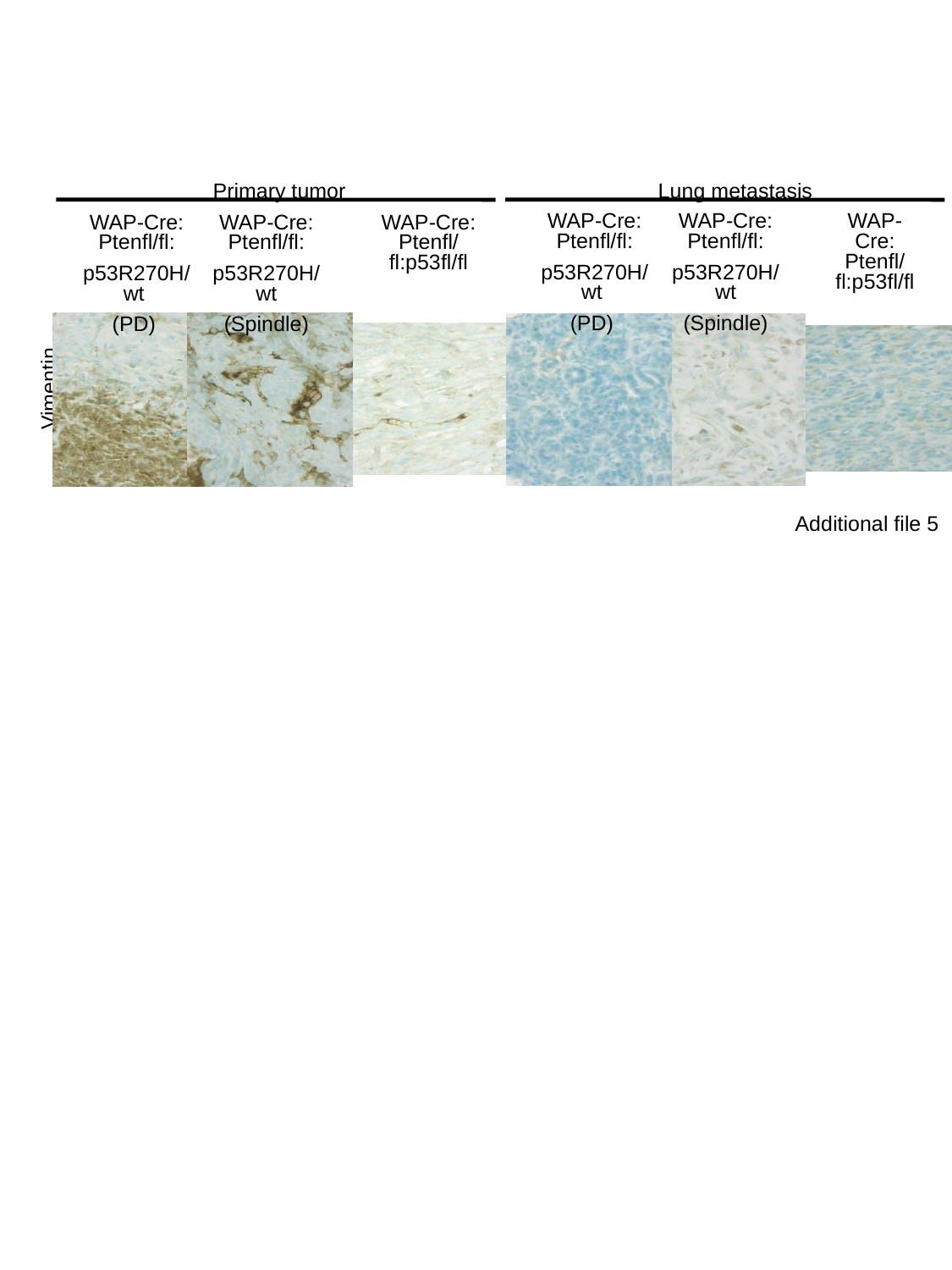

Primary tumor
Lung metastasis
WAP-Cre: Ptenfl/fl:
p53R270H/wt
(PD)
WAP-Cre: Ptenfl/fl:
p53R270H/wt
(Spindle)
WAP-Cre:
Ptenfl/fl:p53fl/fl
WAP-Cre: Ptenfl/fl:
p53R270H/wt
(PD)
WAP-Cre: Ptenfl/fl:
p53R270H/wt
(Spindle)
WAP-Cre:
Ptenfl/fl:p53fl/fl
Vimentin
# Additional file 5
